# Novel Role of ER Stress and Autophagy in Microcystin-LR Induced Apoptosis in Chinese Hamster Ovary Cells

**DOI:** 10.3389/fphys.2016.00527

**Published:** 2016-11-08

**Authors:** Shenshen Zhang, Chuanrui Liu, Yang Li, Mustapha U. Imam, Hui Huang, Haohao Liu, Yongjuan Xin, Huizhen Zhang

**Affiliations:** Department of Environmental Health, College of Public Health, Zhengzhou UniversityZhengzhou, China

**Keywords:** Microcystin-LR (MC-LR), endoplasmic reticulum stress (ERs), autophagy, apoptosis, Chinese hamster ovary (CHO) cells

## Abstract

Microcystin-LR (MC-LR) is a ubiquitous peptide that exhibits strong reproductive toxicity, although the mechanistic basis for such toxicity remains largely unknown. The present study was conducted to investigate the mechanisms underlying the adverse effects of exposure to MC-LR in Chinese hamster ovary (CHO) cells. The results showed that MC-LR inhibited the *in vitro* proliferation of CHO cells significantly, with an IC_50_ of 10 μM. Moreover, MC-LR-treated CHO cells revealed strong induction of cell cycle arrest and apoptosis. Additionally, exposure of CHO cells to MC-LR resulted in excess reactive oxygen species production and intracellular calcium release, with resultant endoplasmic reticulum stress (ERs). There was also extensive accumulation of autophagic vacuoles with the highest concentration of MC-LR used (10 μM). Furthermore, the expression of ERs (GRP78, ATF-6, PERK, IRE1, CHOP) and autophagy (Beclin1 and LC3II) proteins was increased, with concomitantly reduced expression of LC3I suggesting that ERs and autophagy were induced in CHO cells by MC-LR treatment. Conversely, pretreatment of CHO cells with 4-Phenyl butyric acid, the ERs inhibitor reduced the MC-LR-induced apoptotic cell death and cellular autophagy as evidenced by the reduced expression of Beclin1 and LC3II. Similarly, MC-LR treatment in combination with an autophagy inhibitor (3-methyladenine) increased apoptotic cell death compared with MC-LR alone, and induced ERs *via* upregulating ERs proteins. The overall results indicated that activation of ERs and autophagy are both associated with MC-LR-induced apoptosis in CHO cells. ERs may be a trigger of autophagy in this process.

## Introduction

Public health concerns on toxic cyanobacteria have continually increased worldwide in recent years, due to the occurrence of cyanotoxins in freshwater, brackish and marine ecosystems. Cyanotoxins are not only harmful to aquatic biota but also present a threat to human health. Microcystins (MCs) are ubiquitous cyanotoxins, which cause acute hepato- and neuro-toxicity, kidney impairment and gastrointestinal disorders (Rastogi et al., 2014). So far, nearly 90 variants of MCs have been identified with distinct toxicity degrees (Codd et al., 2005; van Apeldoorn et al., 2007). Microcystin-LR (MC-LR) is the most toxic and commonly-occurring variant of MCs that can accumulate in the gonads of embryos and adults, kidneys, liver, heart and brain, eventually causing organ damage (Pearson et al., 2010; Zeng et al., 2014). Recently, increasing studies have also demonstrated that the gonads are the second most affected organs in MC-LR toxicity after the liver (Ding et al., 2006). This can have huge consequences on the reproductive system especially in view of the aggravated pollution of the environment by ubiquitous toxins like MC-LR.

The toxic effects of MC-LR have been demonstrated in a wide variety of animal models including mice, zebrafish, rat and other fish species (Li et al., 2008; Zhang et al., 2008; Papadimitriou et al., 2010; Wu et al., 2014; Zhao et al., 2015; Hou et al., 2016). Furthermore, apoptosis of the testes through the mitochondrial and endoplasmic reticulum (ER) pathways have been implicated in MC-LR-induced reproductive toxicity in frogs (Zhang et al., 2013), while damage to the placental barrier is thought to underlie its maternal and embryonic toxicity in mice (Bu et al., 2006). We have also previously demonstrated that ROS production and apoptosis rate were increased significantly in Chinese hamster ovary (CHO) cells and human bronchial epithelial cells treated with MC-LR, which may contribute to reproductive and respiratory toxicity, respectively (Li et al., 2015). In addition, we demonstrated increased tendency of sertoli cells to undergo apoptosis through mitochondria-dependent pathway following MC-LR-induced increases in ROS production and expression of caspase-9 and -3 (Xue et al., 2015). Increased ROS production, mitochondrial membrane potential (MMP) and intracellular free Ca^2+^ have similarly been demonstrated in rats spermatogonia after exposure to MC-LR (Zhou et al., 2012).

Apoptotic cell death pathways are activated by a diverse array of extrinsic and intrinsic cellular signals. Emerging evidence suggests that chronic or unresolved perturbations in ER function in the form of “ER stress” (ERs) can lead to widespread pathologic apoptosis (Kaufman, 2002). Calcium release from the ER and redox state, for example, has been implicated as key signaling events in ER-stress-induced apoptosis (Tabas and Ron, 2011). Qin et al. reported that MC-LR significantly upregulated the expression of C/EBP homologous protein (CHOP) and cleaved caspase-12 proteins in the liver, and down-regulated Bcl-2 mRNA expression, thus indicating the involvement of ERs pathway in MC-LR-induced apoptosis (Qin et al., 2010). In huh7 cells, the mRNA expression of different key ERs markers including *BiP, ATF-4* and spliced *XBP-1* mRNA were increased, with concomitant increase in the expression of apoptosis-related genes like CHOP and the cytoprotective chaperone BiP (Christen et al., 2013).

Autophagy is an essential self-destructive mechanism by which cells break down their own cellular proteins and organelles in response to various adverse conditions or stress (Kabeya et al., 2000). Among the proteins involved in autophagy, the soluble LC3 is essential for the later formation of autophagosomes (Tanida et al., 2004). The cytoplasmic form of this protein (LC3I) is conjugated to phosphatidylethanolamine to form the LC3-phosphatidylethanolamine conjugate (LC3II) (Barth et al., 2010), which is often used as an indicator to monitor autophagy. LC3 was found to increase at relatively low MC-LR concentrations, while 3-methyladenine (3-MA), an autophagy, attenuated the MC-LR-induced LC3 increase with consequent attenuation of autophagosome accumulation and apoptosis (Chen et al., 2013). Based on previous findings, ERs and autophagy seem to play crucial roles in MC-LR-induced apoptosis and reproductive toxicity. However, the role and mechanisms of ERs and autophagy in apoptosis of CHO cells induced by MC-LR remains to be further explored.

The purpose of the present study was to investigate whether MC-LR could regulate autophagy and ERs, and elicit apoptosis in CHO cells. For mechanistic insights, several protein markers involved in these pathways were detected. Moreover, specific inhibitors were used to investigate the interaction between autophagy and ERs in MC-LR-induced apoptosis in CHO cells.

## Materials and methods

### Chemicals

Microcystin-LR (MC-LR) (purity ≧ 95%, by HPLC) was purchased from Express Technology Co., Ltd (Beijing, China). RPMI 1640 culture medium and fetal bovine serum (FBS) were purchased from Gibco (Grand Island, NY, USA), while 4-Phenyl butyric acid (4-PBA) and 3-MA autophagy inhibitor were purchased from Sigma-Aldrich Inc. (St. Louis, MO, USA). Cell Counting Kit-8 was purchased from Dojindo Lab (Kumamoto, Japan). Reactive oxygen species assay kit and Annexin V-FITC apoptosis detection kit were purchased from Beyotime Biotechnology Company (Nanjing, China). All other reagents were of analytical grade.

### Cell line culture

The CHO cell line was obtained from the Laboratory of Toxicology, Henan Tobacco Research Institute as a gift and grown in RPMI 1640 media supplemented with 10% FBS, 2 mM L-glutamine (Solarbio, Beijing, China), 5 mM HEPES buffer (pH 7.4) (Gibco, NY, USA), 100 U/mL penicillin, and 100 μg/mL streptomycin (Gibco, Grand Island, NY, USA). CHO cells were maintained in a humidified incubator with 5% CO_2_ at 37°C. For assays involving MC-LR, it was dissolved in methanol to prepare stock solution (1 mg/mL) and diluted to the required concentration in PBS. Final concentration of methanol in CHO cells exposed to MC-LR solution was less than 0.01%. For some assays, CHO cells were pretreated with 3-MA (5 mmol/L) or 4-BPA (5 mmol/L) followed by MC-LR solution.

### CCK8 assay for cytotoxicity assessment

Chinese hamster ovary (CHO) cells were seeded in 96-well plates at a density of 2.0 × 10^4^ cells per well and allowed to adhere and grow for 24 h. The culture medium was then replaced by fresh medium containing MC-LR (1–30 μM) or vehicle for another 24 h. Thereafter, CCK-8 solution was added to each well and cytotoxicity was analyzed according to manufacturer's instructions.

### Cell cycle analysis

Chinese hamster ovary (CHO) cells were plated in 6-well plates at 1.0 × 10^6^ cells per well. After incubation for cell adherence and growth, increasing concentrations of MC-LR solution or vehicle were added into corresponding wells. Following incubation for 24 h, CHO cells were harvested and single cell suspension was prepared by trypsinization. After washing twice with cold PBS, cells were fixed with 70% ethanol overnight at 4°C, and then washed with PBS twice again. Propidium iodide (PI) staining solution and RNase A stock solution were added to the cells for 30 min at 37°C in the dark. Cell cycle analysis was done using BD Accuri C6 flow cytometer (BD Biosciences, San Jose, CA, USA) and cells in G_0_/G_1_, S, G_2_/M phases were quantified.

### Analysis of apoptotic cells

Apoptotic cells were determined according to the Annexin V-FITC apoptosis detection kit protocol. After the desired treatments, CHO cells were harvested, washed twice with cold PBS, and then stained with Annexin V-FITC/PI according to the recommended protocol. Apoptotic cells were detected using flow cytometer.

### ROS content measurement

Intracellular ROS were detected using the florescent probe 2′, 7′-dichlorofluorescein diacetate (DCFH-DA). In brief, CHO cells were exposed to MC-LR for 24 h, and then loaded with 10 μM DCFH-DA for 20 min at 37°C in the dark. CHO Cells were harvested and washed to remove the extracellular extra DCFH-DA. ROS were detected by measuring the fluorescence intensity on a flow cytometer.

### Calcium measurement

Fluo 3 AM is a long wavelength calcium probe that is practically nonfluorescent in its free ligand form, but its fluorescence increases 60–100 times when it forms complexes with calcium. This method has been extensively used in Ca^2+^ measurement. Briefly, CHO cells were exposed to different concentrations of MC-LR (0, 2.5, 5, 10 μM), and then loaded with the Ca^2+^ fluorescent probe fluo-3 AM (Sigma, St. Louis, MO, USA) for 30 min at 37°C. After washing, cells were used for fluorescent Ca^2+^ detection on a flow cytometer.

### Detection of autophagic vacuoles by MDC staining

Monodansylcadaverine (MDC) is the autofluorescent dye for labeling of autophagic vacuoles in cells, which accumulates and fluoresces inside membrane compartments. After exposure to MC-LR (0, 2.5, 5, 10 μM), CHO cells were stained with 50 mM MDC (Sigma, St. Louis, MO, USA) in PBS for 15 min, washed twice with PBS, and then observed by a fluorescent microscope. To quantify the MDC in the CHO cells, single CHO cells suspension were prepared by trypsinization and detected on a flow cytometer.

### Western blot analysis

Following the desired treatments, CHO cells were washed and harvested on ice, and then lyzed with ice-cold protein extraction buffer (Beyotime, Nanjing, China). The protein concentration was determined using BCA protein assay kit (ComWin, Beijing, China). Equal amounts of protein were loaded into gel wells, separated by electrophoresis on SDS-polyacrylamide gels and transferred onto PVDF transfer membrane (Millipore, Bedford, MA). The transferred blots were blocked with blocking agents (5% w/v BSA and 0.05% Tween-20 in TBS) for 1 h at room temperature. Blots were then incubated in a certain proportion of specific primary antibodies: anti-GRP78 (ab25192 Rabbit monoclonal, Abcam), anti-ATF-6 (ab203119 Rabbit polyclonal, Abcam), anti-PERK (C33E10 Rabbit polyclonal, Abcam), anti-IRE1 (ab37073 Rabbit polyclonal, Abcam), anti-CHOP (ab11419 Mouse monoclonal, Abcam), anti-Beclin1 (ab55878 Rabbit polyclonal, Abcam), anti-LC3B (ab81785 Rabbit polyclonal, Abcam) (1:1000) overnight at 4°C. These membranes were rinsed 3 times for 10 min each with TBS-Tween (Sigma, St. Louis, MO, USA) and incubated with HRP conjugated secondary antibodies (1:5000) (CWBio, Beijing, China). After washing, protein bands were visualized by an enhanced chemiluminescence detection kit (ComWin, Beijing, China). Each band density was quantified using Image J software (GE Healthcare, Piscataway, New Jersey, USA).

### Statistical analysis

The data are expressed as mean ± standard deviation (SD). Statistical differences between groups were determined using one-way analysis of variance followed by a Student-Newman-Keuls (SNK) test using SPSS 21.0. The value *P* < 0.05 was regarded as statistically significant.

## Results

### MC-LR inhibited the proliferation of CHO cells

**Figure 1:**
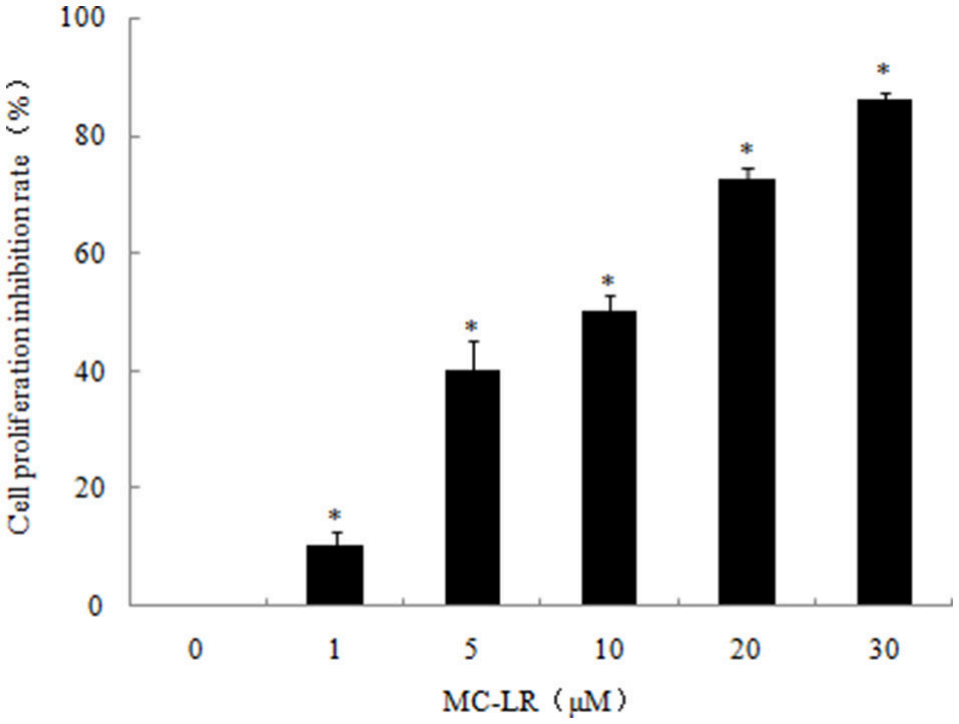
**Dose-dependent inhibition of Chinese hamster ovary (CHO) cell growth by Microcystin-LR (MC-LR)**. CHO cells were exposed to the indicated concentrations of MC-LR or vehicle, and then incubated for 24 h. The relative cell viabilities were tested using CCK8 assay. The data are representative of three independent experiments and expressed as the means ± SD; ^*^P < 0.05 significantly different from vehicle control group.

CCK8 assay was conducted to investigate the growth-inhibitory effects of MC-LR against CHO cells. As shown in Figure 1, treatment of CHO cells with increasing concentrations of MC-LR (1 to 30 μM) for 24 h elicited dose-dependent decreases in cell viability compared to the vehicle control, suggesting cytotoxicity and/or anti-proliferation by MC-LR. The IC_50_ value of MC-LR for CHO cell line was 10 μM, hence IC_50_/4, IC_50_/2, and IC_50_ concentrations (2.5, 5, 10 μM, respectively) were used in subsequent experiments.

### MC-LR induced G_2_/M arrest in CHO cells

**Figure 2:**
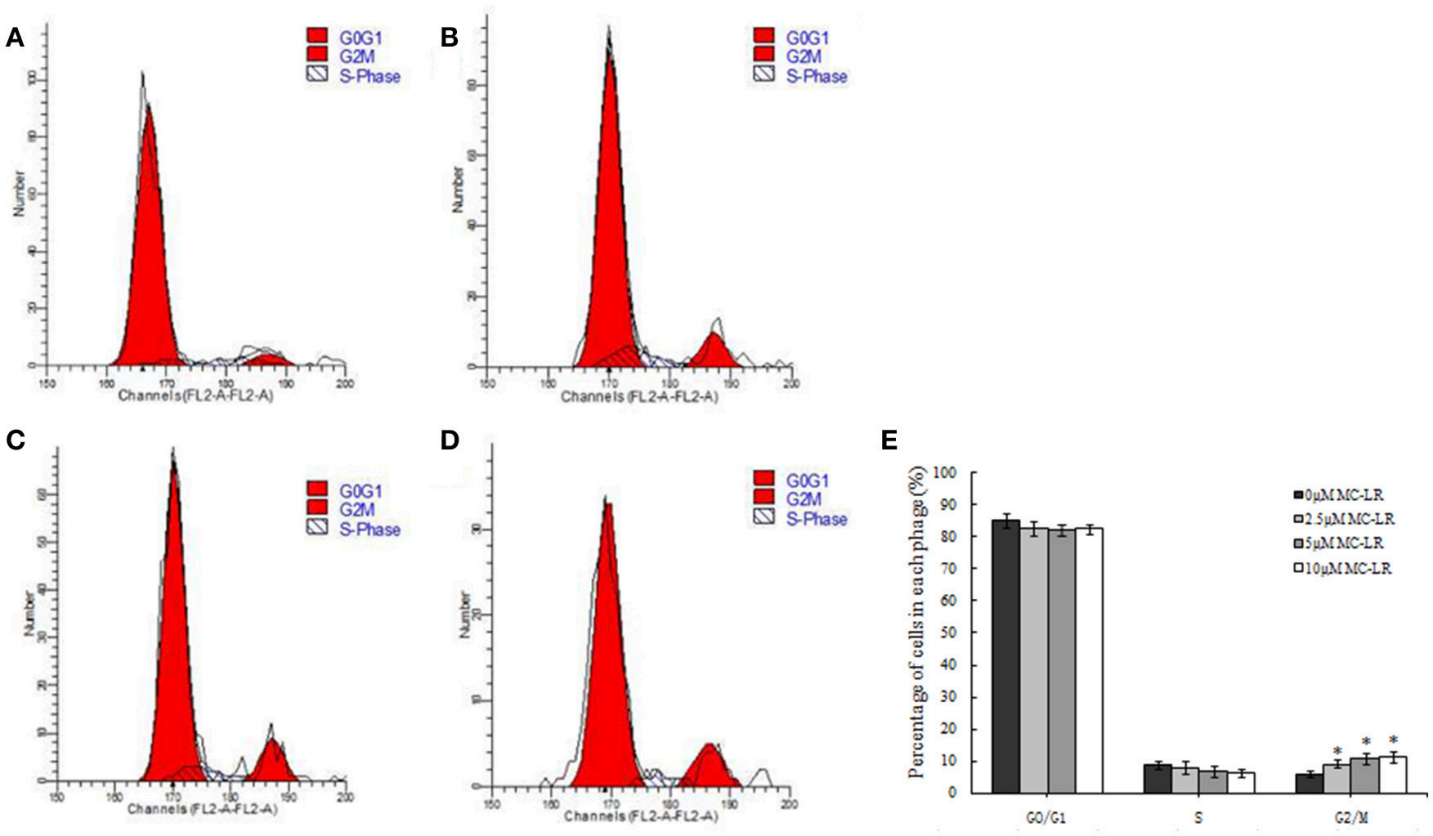
**G_**2**_/M phage arrest by Microcystin-LR (MC-LR) treatment in Chinese hamster ovary (CHO) cells**. CHO cells were treated with 0–10 μM MC-LR for 24 h, and the cell cycle phase distribution was determined using flow cytometry. **(A)** 0 μM MC-LR, **(B)** 2.5 μM MC-LR, **(C)** 5 μM MC-LR, **(D)** 10 μM MC-LR, **(E)** Data are the means ± SD of three independent experiments. ^*^P < 0.05 vs. the vehicle control.

As shown in Figure 2, cell cycle analysis revealed that CHO cells arrested in the G_2_/M phase of the cell cycle in the presence of MC-LR. The proportion of cells in the G_2_/M phase was increased to 11.78% for the 10 μM MC-LR groups, respectively, compared to the vehicle control group (6.08%). The proportion of cells was correspondingly decreased in the S phase. These findings were consisted with our previous results (Li et al., 2014). It suggested that MC-LR induced CHO cells arrest in the G_2_/M phase and inhibited cell growth.

### Effects of MC-LR on CHO cells apoptosis

**Figure 3:**
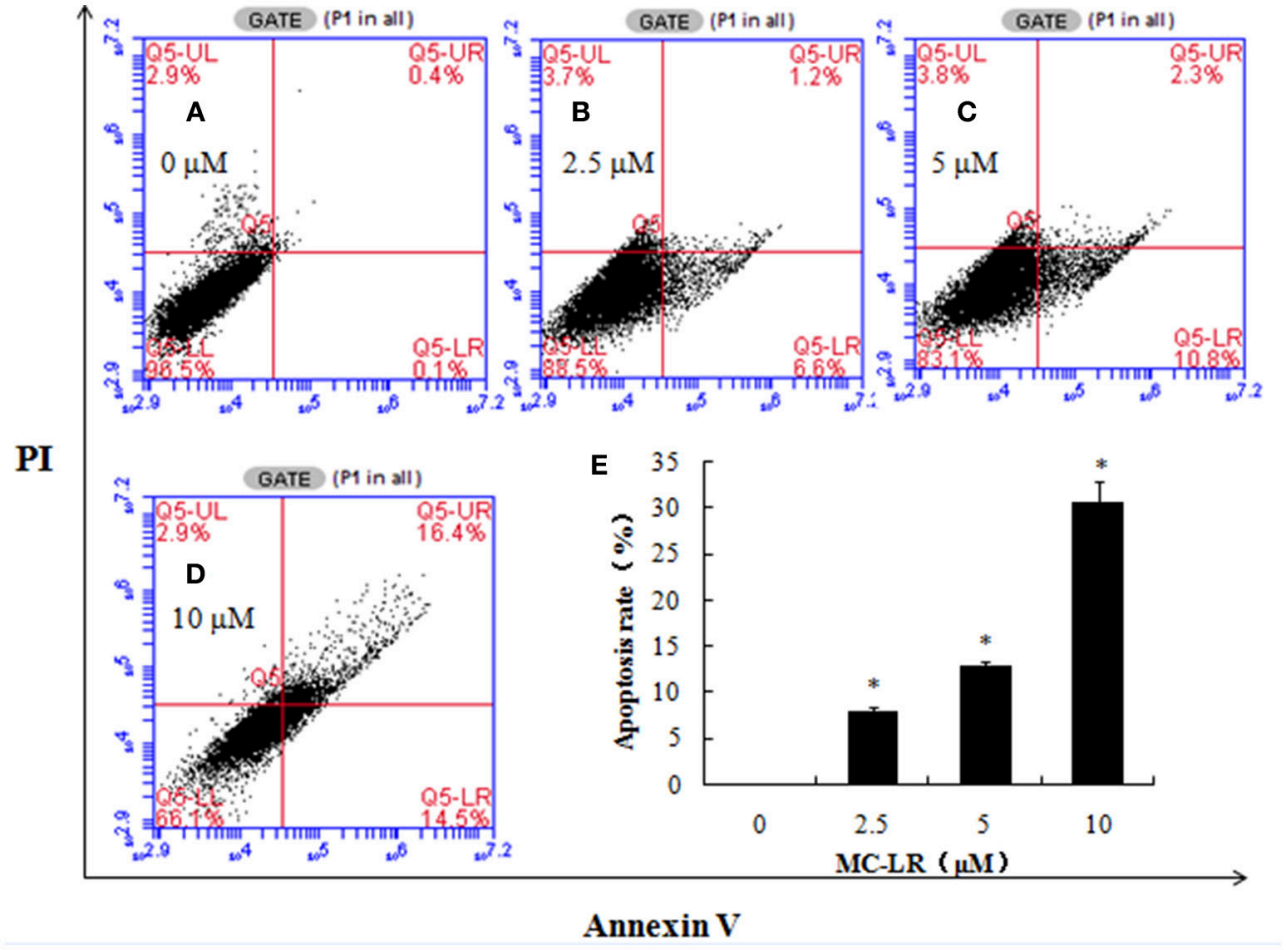
**Microcystin-LR (MC-LR) induces apoptotic cell death in Chinese hamster ovary (CHO) cells**. CHO cells were cultured in the presence of the desired concentrations of MC-LR and subjected to flow cytometry. **(A)** 0 μM MC-LR, **(B)** 2.5 μM MC-LR, **(C)** 5 μM MC-LR, **(D)** 10 μM MC-LR, **(E)** Bars represent means ± SD of three independent experiments. (^*^) Denotes significantly different means compared to the vehicle control (^*^P < 0.05).

The externalization of phosphatidylserine is one of the leading indicators of apoptosis, while the incorporation of PI is indicative of cell necrosis. We used flow cytometry to investigate the extent to which MC-LR caused apoptosis and/or necrosis in CHO cell cultures. Figure 3 shows dose-dependent cytotoxicity in CHO cells after 24 h exposure to MC-LR. There were 16.4% of cells in late apoptosis/necrosis stage in the 10 μM MC-LR-treated CHO cells vs. 0.4% of vehicle control. These results suggested that apoptosis was induced by MC-LR.

### Effects of MC-LR on intracellular ROS levels in CHO cells

**Figure 4:**
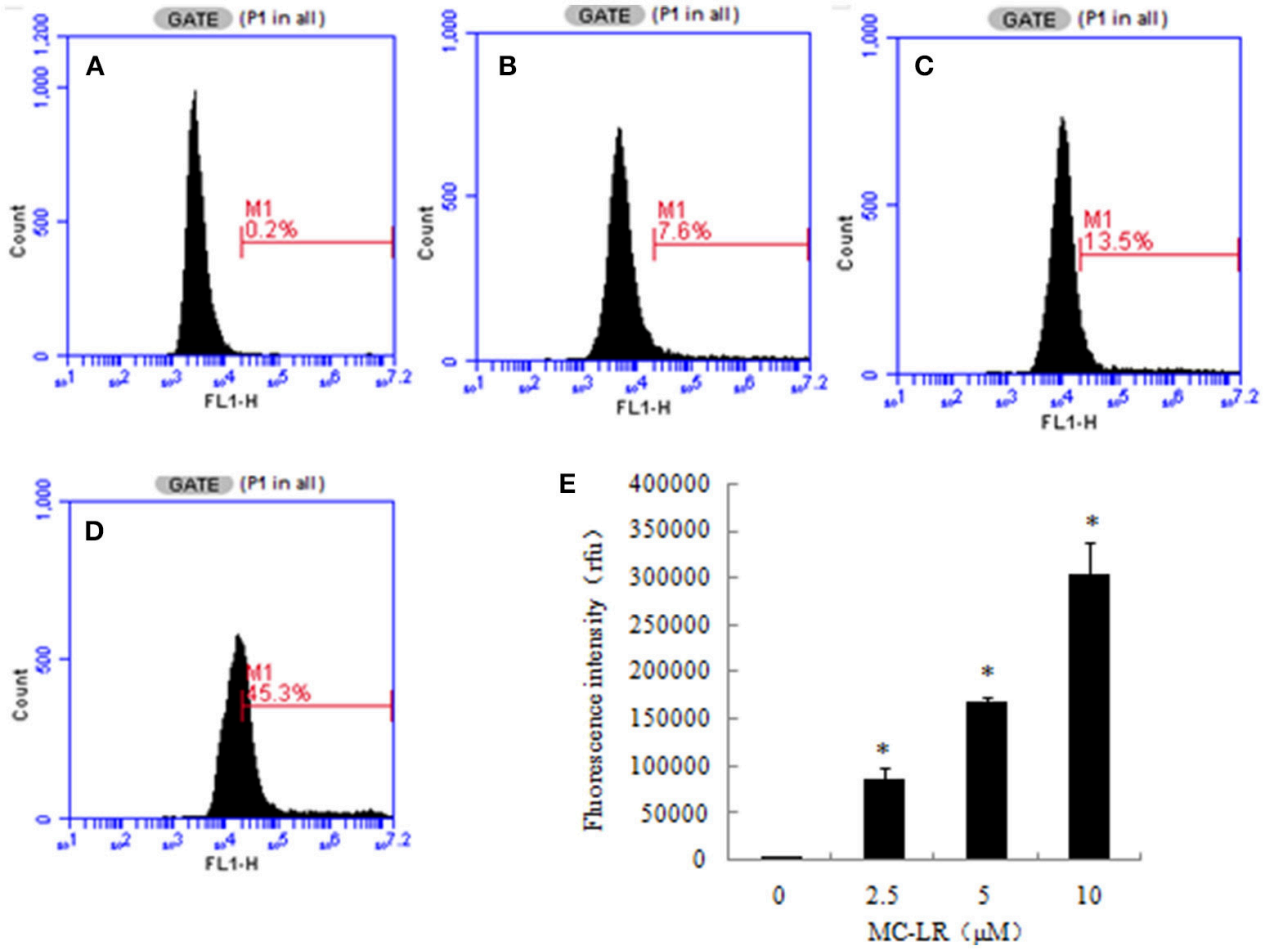
**Microcystin-LR (MC-LR) increased reactive oxygen species (ROS) production in Chinese hamster ovary (CHO) cells. (A–D)** Cells treated with different concentrations of MC-LR for 24 h, **(A)** 0 μM, **(B)** 2.5 μM, **(C)** 5 μM, **(D)** 10 μM. The intracellular ROS levels were measured using flow cytometry. **(E)** Bars present means ± SD of three independent experiments. ^*^P < 0.05 vs. vehicle control.

ROS production is a critical indicator of cell apoptosis. In this study, ROS production in CHO cells was determined through flow cytometric analysis. Higher ROS levels were observed in the MC-LR-treated CHO cells compared to the vehicle control group. The intracellular ROS levels represented by the DCF fluorescence intensity increased dose-dependently following MC-LR treatment, which was upregulated from 4120.00 (0 μM) to 290262.66 (10 μM) (Figure 4).

### Detection of intracellular Ca^2+^ concentration after exposure to MC-LR in CHO cells

**Figure 5:**
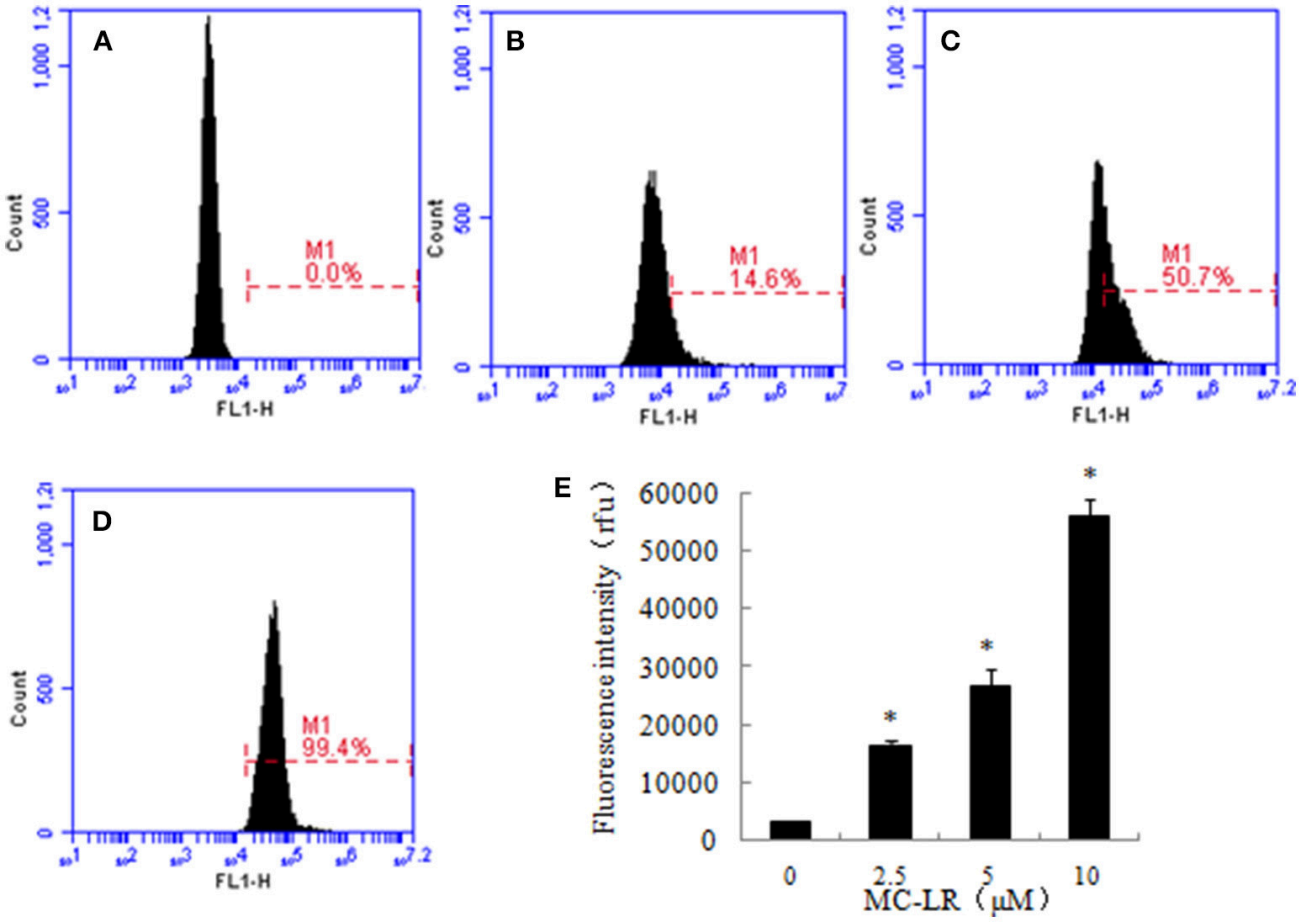
**The effect of Microcystin-LR (MC-LR) on intracellular Ca^**2+**^ concentration**. Intracellular Ca^2+^ levels were measured in different groups by Fluo-3/AM fluorescent probes through flow cytometry. **(A)** 0 μM, **(B)** 2.5 μM, **(C)** 5 μM, **(D)** 10 μM, **(E)** Bars represent the mean values of three replications ± SD. ^*^P < 0.05 compared to vehicle control.

Intracellular Ca^2+^ storage sites include the mitochondria and endoplasmic reticulum, and their alterations are known to be involved in the process of apoptosis and ERs. We investigated whether MC-LR alters intracellular Ca^2+^ using fluo-3/AM fluorescent probes. The results demonstrated that MC-LR induced a marked elevation of fluorescence intensity after exposure to MC-LR (Figure 5). The fluorescence intensity of the 10 μM MC-LR group increased by around 18 times compared to the untreated CHO cells. The results suggested that intracellular Ca^2+^ release may be involved in MC-LR-induced CHO cells apoptosis.

### Detection of autophagic vacuoles in CHO cells

**Figure 6:**
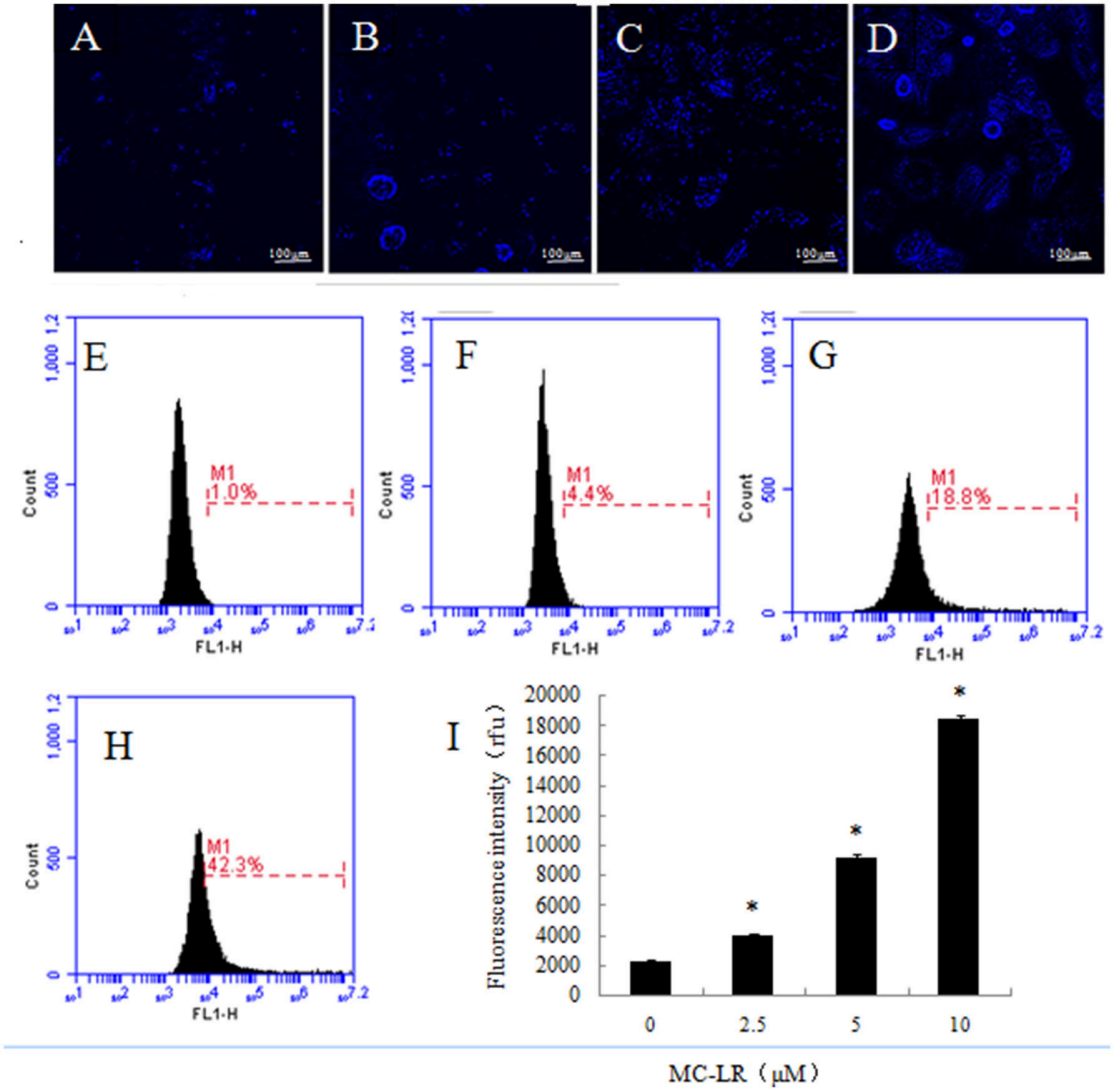
**Detection of autophagic vacuoles in Chinese hamster ovary cells**. **(A–D)** Cells were incubated for 24 h with increasing concentrations of MC-LR and then stained by monodansylcadaverine, after which fluorescence imaging (400×) was accomplished by live cell imaging system. **(A)** 0 μM, **(B)** 2.5 μM, **(C)** 5 μM, **(D)** 10 μM. **(E–H)** Autophagy was evaluated using monodansylcadaverine staining. **(E)** 0 μM, **(F)** 2.5 μM, **(G)** 5 μM, **(H)** 10 μM, **(I)** Bars represent mean ± SD of three independent experiments. ^*^P < 0.05 compared with the vehicle control group.

Autophagic vacuoles are hallmarks of autophagy. In our study, live cell imaging system and flow cytometry were employed to analyze the presence of autophagic vacuoles in CHO cells, which were observed after exposure to MC-LR. Compared to the vehicle control group (0 μM), both fluorescence intensity and the number of autophagic vacuoles increased remarkably (Figures 6A–D). This phenomenon was, to some extent, supported by flow cytometry in which the fluorescence intensity of the 10 μM MC-LR group was increased by ~7 fold (Figures 6E–I). The results suggested that MC-LR induced autophagic vacuoles formation in CHO cells.

### MC-LR treatment regulates the levels of ERs- and autophagy-related proteins in CHO cells

**Figure 7:**
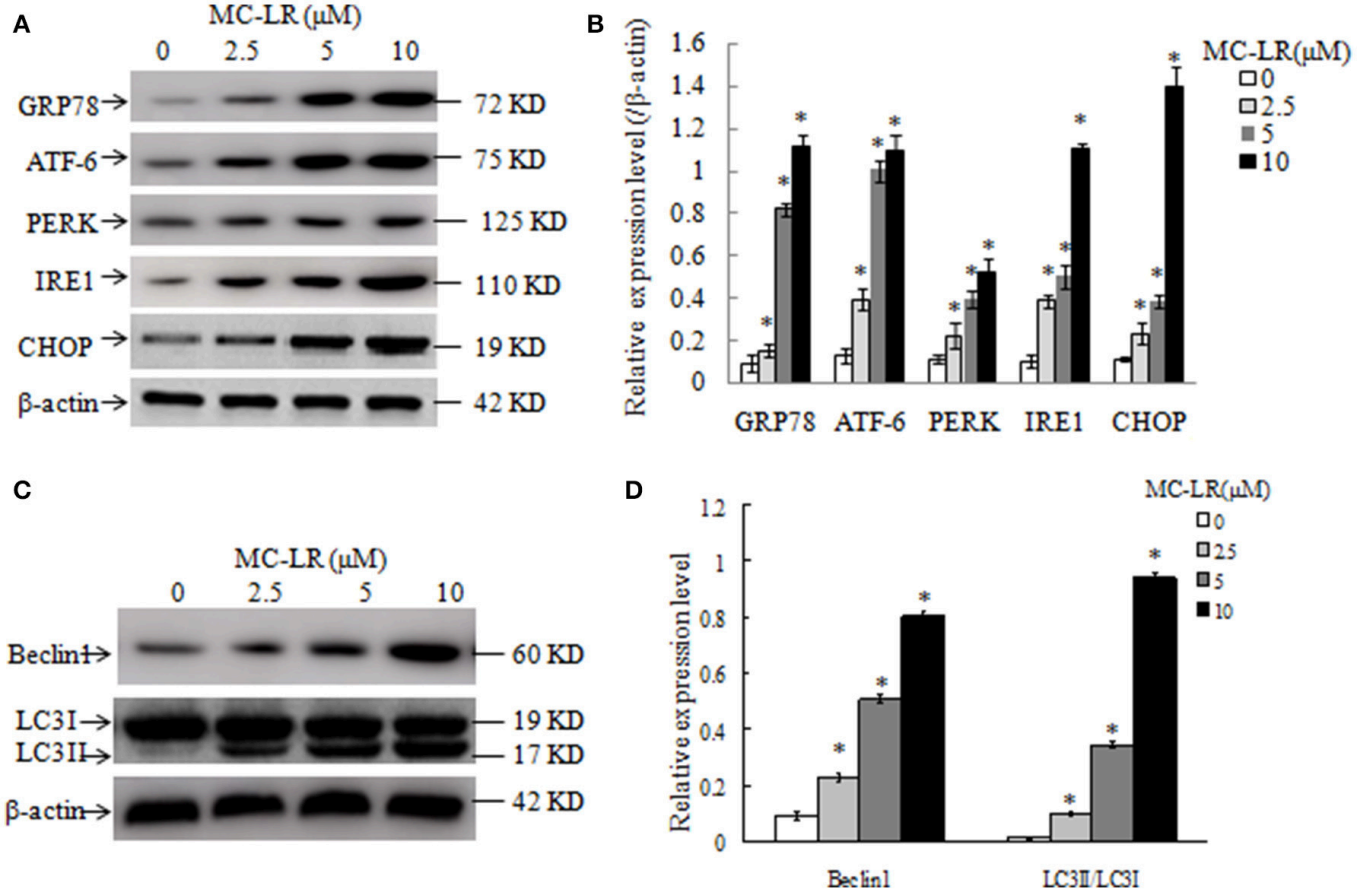
**Western blotting of endoplasmic reticulum stress- and autophagy-related proteins in Chinese hamster ovary (CHO) cells**. Western blotting of GRP78, ATF-6, PERK, IRE1, CHOP **(A)**, Beclin1, LC3II and LC3I **(C)** in CHO cells after treatment with MC-LR at various concentrations for 24 h. The percentage of proteins was normalized to β-actin and expressed as the average of three independent experiments ± SD **(B,D)**. ^*^P < 0.05 compared with the vehicle control group.

To further clarify the molecular mechanisms by which MC-LR induced cell apoptosis, we tested the effects of MC-LR on the expression of ERs- and autophagy proteins *via* western blotting. GRP78, ATF-6, PERK, IRE1, and CHOP are biomarkers of ERs in mammalian cells. Figures 7A,B shows that the expression levels of GRP78, ATF-6, PERK, IRE1, and CHOP were enhanced in MC-LR-treated CHO cells. The conversion of soluble LC3-I to lipid bound LC3-II is associated with the formation of autophagic vacuoles and completion of autophagy (28). Beclin-1 is also a critical component in the process of autophagic vacuole formation. As shown in Figures 7C,D, expression of LC3-II and Beclin-1 was markedly elevated due to MC-LR treatment. As expected, LC3-I expression decreased in the presence of MC-LR owning to conversion to LC3-II, with a significant upregulation of the ratio of LC3-II/LC3-I. These results indicated that MC-LR treatment induced ERs and autophagy activation in CHO cells.

### Inhibition of apoptosis in CHO cells through ERs- and autophagy-dependent signaling pathway

**Figure 8:**
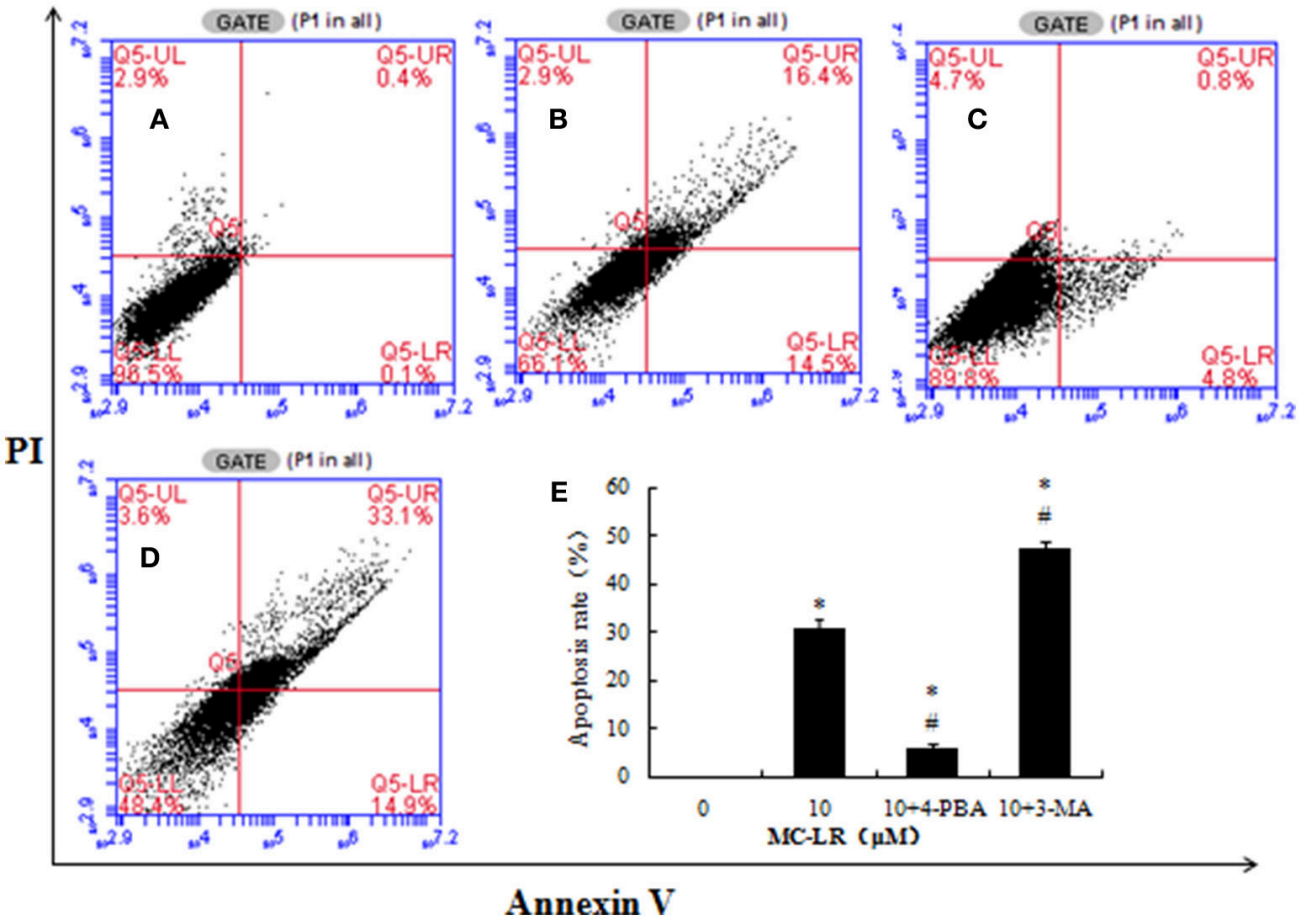
**Effects of Microcystin-LR (MC-LR) on apoptotic cell death in Chinese hamster ovary (CHO) cells after pre-treatment with 4-PBA (5 mmol/L) or 3-MA (5 mmol/L)**. CHO cells were pre-treated with 4-PBA or 3-MA and then co-cultured with 10 μM MC-LR for 24 h. Cells were then stained with Annexin V-FITC/PI and measured by flow cytometry. **(A)** 0 μM MC-LR, **(B)** 10 μM MC-LR, **(C)** 10 μM MC-LR + 4-PBA, **(D)** 10 μM MC-LR + 3-MA, **(E)** Values are expressed as the means ± SD of three independent experiments. (^*^) *P* < 0.05 vs. Vehicle control group (0 μM MC-LR), (^#^) *P* < 0.05 vs.10 μM MC-LR.

To investigate the role of ERs and autophagy in MC-LR-induced apoptosis, a specific ERs inhibitor (4-PBA) or autophagy inhibitor (3-MA) was used to pre-treat CHO cells prior to MC-LR treatment. Based on the above results, 10 μM of MC-LR was used in the related experiment because of the most obvious toxic effects on CHO cells. MC-LR-induced CHO cell apoptosis was significantly inhibited by the combination of 4-PBA + MC-LR compared to MC-LR only (Figure 8). On the contrary, autophagy inhibitor 3-MA combined with MC-LR significantly promoted the apoptosis of CHO cells, leading to much higher cell apoptosis rate than in the MC-LR only treatment group.

### Inhibition of autophagy stimulates ERs in CHO cells

**Figure 9:**
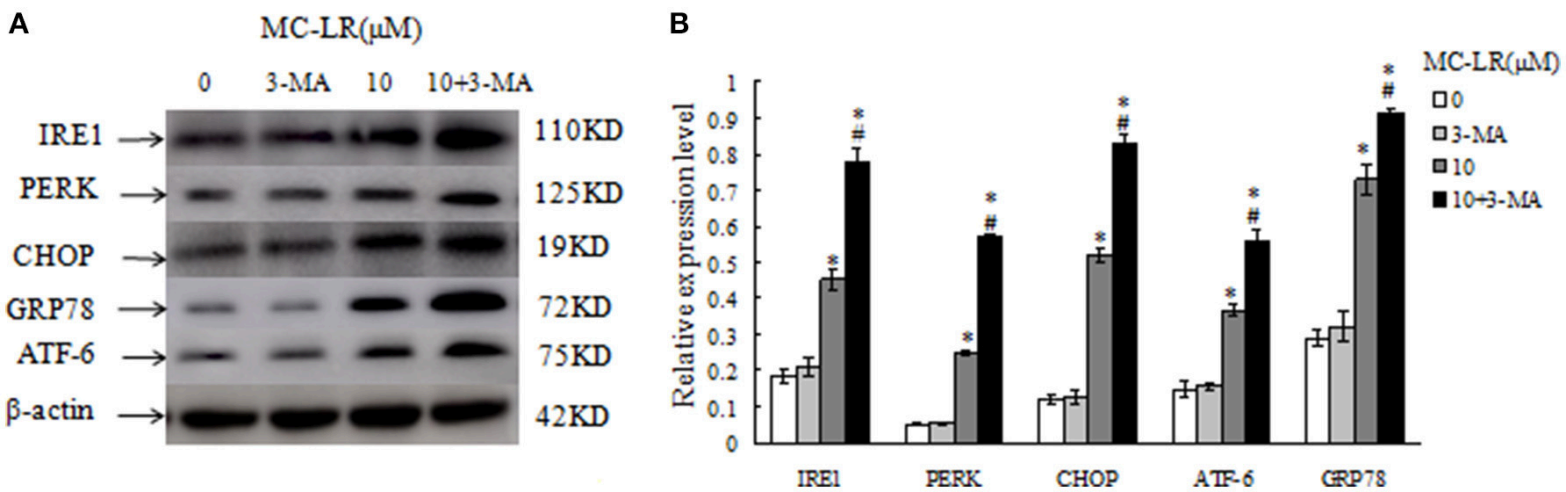
**Western blotting of endoplasmic reticulum stress biomarkers in Chinese hamster ovary (CHO) cells, in the presence or absence of 3-MA (5 mmol/L) after treatment with Microcystin-LR (MC-LR). (A)** Western blotting of GRP78, ATF-6, PERK, IRE1, and CHOP in CHO cells after treatment with MC-LR in the presence or absence of 3-MA. **(B)** Quantitation of protein expressions. The ratio of target protein expression is normalized to internal control (β-actin) in CHO cells from corresponding group, (^*^) *P* < 0.05 in comparison with vehicle control group, (^#^) *P* < 0.05 in comparison with 10 μM MC-LR (mean ± SD, *n* = 3).

Chinese hamster ovary (CHO) cells were pretreated with autophagy inhibitor 3-MA to block autophagy prior to MC-LR treatment. Western blotting analysis indicated that the expression levels of ERs biomarker proteins, GRP78, ATF-6, PERK, IRE1, and CHOP, were upregulated after 10 μM of MC-LR treatment; 3-MA treatment alone did not affect the biomarkers expression significantly compared to control group. Moreover, the levels of these biomarkers was further increased in the 3-MA + MC-LR group compared with the MC-LR group (*P* < 0.05; Figure 9). The results suggested that inhibition of autophagy may stimulate ERs in MC-LR treated CHO cells.

### Inhibition of ERs suppresses autophagy in CHO cells

**Figure 10:**
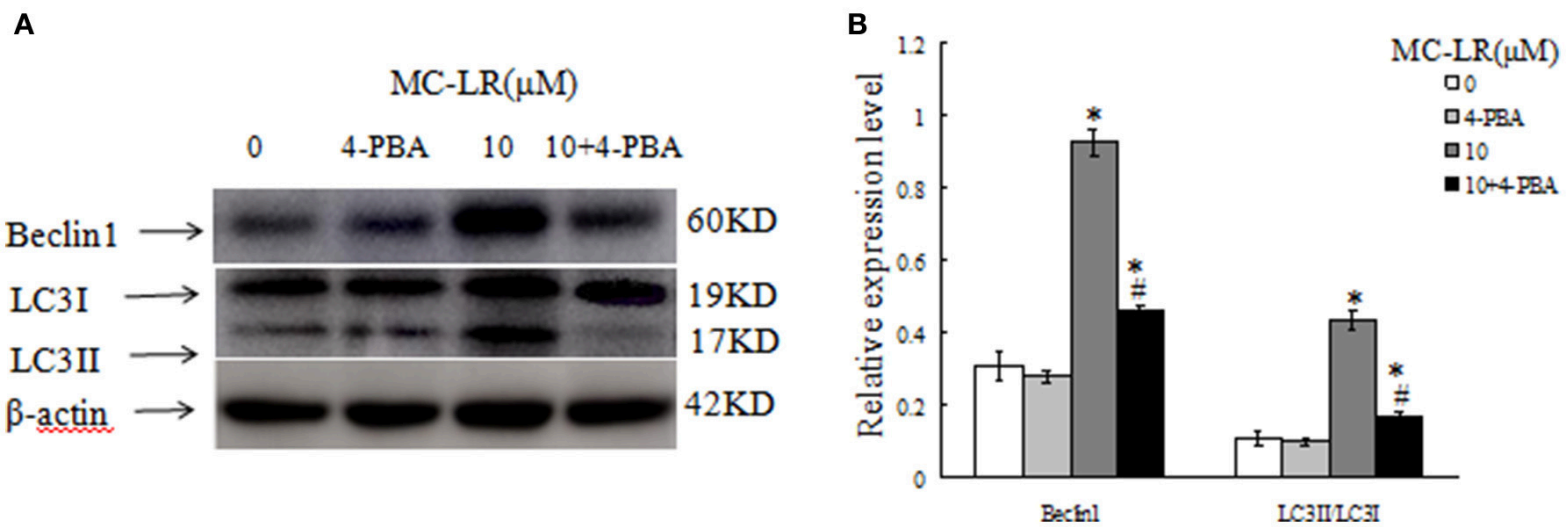
**Western blotting of autophagy-associated proteins, in the presence or absence of 4-PBA (5 mmol/L), after treatment with Microcystin-LR (MC-LR). (A)** Western blotting of Beclin 1, LC3I and LC3II in Chinese hamster ovary cells after treatment with MC-LR in the presence or absence of 4-PBA. **(B)** Quantitation of protein expressions. The ratio of target protein expression is normalized to internal control (β-actin) in CHO cells from corresponding group. (^*^) *P* < 0.05 in comparison with vehicle control group, (^#^) *P* < 0.05 in comparison with 10 μM MC-LR (mean ± SD, *n* = 3).

To investigate the relationship between ERs and autophagy in the MC-LR-induced CHO cell apoptosis, ERs inhibitor 4-PBA or vehicle was added to the corresponding groups before MC-LR treatment. Biomarkers of autophagy activation like Beclin 1 and LC3II were both decreased, whereas LC3I was increased in the 4-PBA + MC-LR group compared with the MC-LR only group. These biomarkers were not significantly influenced by 4-PBA treatment alone (Figure 10). The result indicated that ERs is essential for autophagy activation in MC-LR-induced CHO cells apoptosis.

## Discussion

Toxin-producing cyanobacteria are receiving increasing worldwide attention because they produce hazardous cyanotoxins (MCs). Environmental exposure to MCs causes a variety of serious health issues, including reproductive toxicity with resultant decline in fertility. The gonads are the second largest target organs for MCs after the liver. Our previous studies have demonstrated that one of the most toxic cyanotoxins, MC-LR, induced oxidative injury and apoptosis in CHO cells that are related to the female reproductive system (Li et al., 2015). We also demonstrated that N-acetylcysteine could protect CHO cells from oxidative injury and apoptosis induced by MC-LR (Xue et al., 2015). However, few reports are available about the detailed mechanisms and signaling pathways involved in MC-LR-induced CHO cells apoptosis and decline in female reproductive ability.

In our present study, the results provide some compelling insights into the molecular mechanisms of MC-LR-induced CHO cells apoptosis and damage. Many studies have demonstrated the lethality of MC-LR on various cells including the human liver 7702 cells, rat testicular Sertoli cells and mouse spermatogonia (Chen et al., 2013; Sun et al., 2014; Zhou et al., 2014). Here, we found that exposure to 1~30 μM of MC-LR brought about inhibition of CHO cells growth. In order to choose the optimal concentration of MC-LR for further study, the IC_50_ was calculated to be about 10 μM in CHO cells. Therefore, treatment with 2.5, 5 and 10 μM of MC-LR was finally defined as the appropriate range of concentrations for subsequent experiments. Moreover, these concentrations were consistent with our previous reports (Xue et al., 2015).

The cell cycle is a highly conserved and ordered process in which a cell grows and divides to create a copy of itself. In eukaryotes, the cell cycle is divided into four characteristic phases: mitosis (M), gap 1 (G_1_), DNA synthesis (S), and gap 2 (G_2_). The main checkpoints, G_1_/S, G_2_/M and the metaphase (mitotic) checkpoints, ensure that damaged or incomplete DNA is not passed on to daughter cells. Cell cycle dysregulation always leads to cycle arrest, apoptosis and uncontrolled growth (Wang et al., 2009). MC-LR exposure significantly caused a drastic accumulation in G_0_/G_1_-phase of the cell cycle, leading to G_1/_S arrest and apoptosis of HeLa cells (Chen et al., 2014). Exposure to CHO-K1 cells with MC-LR caused accumulation of abnormal G_2_/M figures and abnormal anaphases (Lankoff et al., 2003). The present study also found that treatment with MC-LR for 24 h potently arrested G_2_ phase of CHO cell cycle. It is suggested that the induction of cell cycle arrest may contribute to MC-LR-induced cell growth inhibition.

Apoptosis is the process of programmed cell death in multicellular organisms, which plays important roles in various processes, such as normal cell turnover, proper development and chemical-induced cell death. However, inappropriate apoptosis triggered by certain drugs and chemicals, radiation and various disease states can lead to neurodegenerative diseases, ischemic damage, autoimmune disorders and many types of cancers (Elmore, 2007). MC-LR-induced apoptosis in mammalian cells has been widely confirmed; MCs exposure can result in an excessive increase in oxidative stress, which can subsequently trigger apoptosis (Amado and Monserrat, 2010). Jiang et al. reported that MC-LR could induce significant apoptosis in carp liver cells after a 12 h exposure (Jiang et al., 2013). Mice treated with sublethal toxin doses revealed a discernible intensity of staining in the centrilobular regions and exhibited high levels of apoptotic cells (Yoshida et al., 1998). We also obtained consistent results that showed significant increase in CHO cells apoptosis after 24 h exposure to MC-LR.

It is well-known that over-production of ROS is a common mechanism that promotes apoptosis. ROS are mainly generated during mitochondrial oxidative metabolism and serve to physiologically regulate normal cell proliferation and differentiation. However, excessive ROS can cause severe damage to DNA and proteins, and cause apoptosis. MC-LR was reported to induce cytotoxic effects in embryo, reproductive system and hepatocytes by inducing mitochondrial production of ROS (Moreno et al., 2005; Weng et al., 2007). In this study, ROS generation in the MC-LR treated groups was significantly enhanced after 24 h exposure. This suggested that the increased ROS may play an important role in the CHO cell apoptosis induced by MC-LR.

Intracellular Ca^2+^ is an essential player in the mechanism of apoptosis (McConkey and Orrenius, 1996). ER is the main Ca^2+^ storage site for the cell, and together with the mitochondria, they regulate Ca^2+^ signaling. When cellular Ca^2+^ channels opened, intracellular Ca^2+^ stores release Ca^2+^ through the plasma membrane leading to high concentrations of cellular Ca^2+^. Prolonged opening of these channels can result in excess intracellular Ca^2+^, which can lead to ERs-induced cell death (Krebs, 1998). In the current investigation, we showed that intracellular Ca^2+^ was profoundly elevated in CHO cells incubated with MC-LR in contrast with the CHO cells cultured with vehicle. Paula Kujbida et al. also demonstrated similar findings for different MCs (MC-LA, MC-YR, and MC-LR), in which the MCs induced intracellular Ca^2+^ mobilization as well as Ca^2+^ influx in to neutrophils (Kujbida et al., 2009). We speculate that the activation of intracellular Ca^2+^ release may result in ERs, with subsequent induction of cell death in CHO cells.

Autophagy is an essential self-destructive homeostatic process for balancing sources of energy by breaking down damaged organelles, as well as eliminating intracellular pathogens (Glick et al., 2010). Additionally, ERs can stimulate autophagy to remove unwanted cellular components (Ogata et al., 2006). Alverca et al. reported that large autophagic vacuoles are assembled in a kidney cell line (Vero-E6) after low toxic MC exposure, indicating that autophagy is an early cellular response to the toxin (Alverca et al., 2009). We also found that more autophagic vacuoles were formed in CHO cells with increasing concentrations of MC-LR, implying that autophagic vacuoles may contribute to reducing and neutralizing the toxin.

Beclin l is critical for the signaling pathway activating autophagy and in the process of autophagosome accumulation (Su et al., 2002). Concomitantly, a cytosolic form of LC3 (LC3-I) is conjugated to phosphatidylethanolamine to form LC3-phosphatidylethanolamine conjugate (LC3-II), which is the most reliable marker of autophagy (Feng et al., 2013). To further confirm the role of autophagy in MC-LR-induced apoptosis, the protein expression of autophagy markers was investigated. Levels of Beclin1 and LC3 II were increased with increasing concentrations of MC-LR. Conversely, LC3I expression was reduced and negatively correlated with MC-LR concentrations.

Endoplasmic reticulum (ER) function can be disturbed by a number of environmental factors, resulting in ERs. During ERs, the unfolded or misfolded proteins trigger the unfolded protein response (UPR), which is mediated by three ER membrane transducers, PERK, ATF-6, and IRE1 (Hetz, 2012). The 78-kDa GRP78 is a chaperon protein and a vital regulator of ERs. In non-stressed cells, GRP78 binds to all three transducers in the inactive state, but is released from these sensors to become activated during ERs, thus triggering the UPR (Lee, 2005). In the present study, we examined the role of ERs in the apoptosis of CHO cells exposed to MC-LR for 24 h. GRP78 expression was enhanced to stabilize protein folding during ERs. Moreover, protein levels of the three transducers, PERK, IRE1, and ATF6, were upregulared in the MC-LR groups, indicating the activation of the pathways they regulate. CHOP is one of the downstream components in the UPR-mediated apoptotic pathway in response to ERs, which leads to apoptotic cell death in a variety of cell types (Tang et al., 2016). We also found that the expression of CHOP protein increased significantly after MC-LR treatment. Thus, we suspected that CHOP induced the pro-apoptotic pathway of the ERs to mediate CHO cell apoptosis under exposure to MC-LR. Therefore, the induction of ERs and cell apoptosis are contributing factors to the toxicity of MC-LR.

Both autophagy and ERs were induced in CHO cells under MC-LR exposure, which may have stimulated apoptosis of the cells. To clarify the mechanisms involved in apoptosis of CHO cells due to autophagic vacuole accumulation and ERs induced by MC-LR, the specific autophagy inhibitor 3-MA, or ERs inhibitor 4-PBA was used with MC-LR in CHO cells. The results showed that inhibiting autophagy activated MC-LR-induced apoptosis cascades in CHO cells, whereas the ERs inhibitor, 4-PBA, suppressed this apoptosis effect.

Several previous studies have investigated the link between ERs and autophagy in cell apoptosis. However, much remains to be explored regarding how ERs activates autophagy and promotes cell apoptosis. Interestingly, studies have shown that both ERs and autophagy are integrated parts of the cell pro-survival mechanisms (Fouillet et al., 2012). We demonstrated that suppressing ERs by the inhibitor 4-PBA resulted in the reduction of the autophagy markers LC3II and Beclin 1, with concomitant increase in LC3I. It is suggested that autophagy activation may depend on ERs induction to protect against apoptosis in CHO cells under MC-LR exposure. We found that ERs is a potent trigger of autophagy, which is consistent with previous findings (Criollo et al., 2007). Moreover, inhibiting autophagy with 3-MA increased the expressions of ERs markers, GRP78, ATF-6, PERK, IRE1, and CHOP, thus activating apoptosis. This indicated that inhibiting autophagy could in turn stimulate ERs under MC-LR treatment.

In summary, our results demonstrated that MC-LR exposure inhibited cell proliferation, induced G_2_/M arrest and caused apoptosis in CHO cells. Both ROS production and Ca^2+^ release were increased in the presence of MC-LR, leading to enhanced ERs and higher cell apoptosis rate. In addition, the self-defense mechanism, autophagy, plays a crucial role in protecting CHO cells from MC-LR-induced apoptosis. The interaction between ERs and autophagy were able to modulate CHO cell apoptosis under MC-LR treatment. Collectively, these results suggested that ERs and autophagy are involved in the MC-LR-induced apoptosis of CHO cells. Targeting ERs and autophagy could be a promising therapeutic strategy for protecting against MC-LR toxicity.

## Author contributions

SZ: Study Design, data interpretation, manuscript preparation, literature search. CL: Data collection, literature search. YL: Data collection, literature search. MI: Data Interpretation, manuscript preparation, HH: Data collection, literature search, HL: Data collection, literature search, YX: Literature search, HZ: Study design, data interpretation, manuscript preparation, and funds collection.

### Conflict of interest statement

The authors declare that the research was conducted in the absence of any commercial or financial relationships that could be construed as a potential conflict of interest.
